# A Case Report of Red Blood Cell Alloimmunization and Delayed Hemolytic Transfusion Reaction in a Patient with an Uncommon Phenotype in Sickle Cell Disease: Review of Diagnosis and Management

**DOI:** 10.1155/2024/9980747

**Published:** 2024-09-19

**Authors:** Cassandra P. Wang, Denise Malicki, Courtney D. Thornburg, Sonya Martinez, Jennifer C. Yu

**Affiliations:** ^1^ Department of Pediatrics Rady Children's Hospital, San Diego, CA, USA; ^2^ Department of Pathology and Laboratory Medicine Rady Children's Hospital, San Diego, CA, USA; ^3^ Department of Pathology Division of Pediatric Pathology University of California, La Jolla, San Diego, CA, USA; ^4^ Division of Pediatric Hematology Oncology Rady Children's Hospital, San Diego, CA, USA

## Abstract

A delayed hemolytic transfusion reaction (DHTR) is a potential complication for patients with sickle cell disease (SCD) who develop red blood cell (RBC) alloimmunization to foreign antigens from allogeneic transfusions, potentially resulting in life-threatening hemolytic anemia between 24 hours and 28 days after the transfusion. Guidelines have suggested obtaining an extended RBC antigen profile by genotyping in patients with SCD to provide increased accuracy for antigen matching. We present a pediatric patient with SCD and a rare RBC phenotype that was not identified by serology who developed DHTR after her second lifetime transfusion and highlight the potential advantages of molecular genotyping. She was successfully managed by transfusion with “least incompatible” packed RBCs and aggressive medical management per American Society of Hematology clinical guidelines. Molecular genotyping is advantageous over serologic phenotyping because it can provide additional antigen information, such as increased accuracy for C antigen determination and Fy^b^ antigen matching. Having RBC genotyping results on file for patients with SCD can facilitate care in two ways—by preventing alloimmunization with potential hemolytic transfusion reaction and by responding rapidly to request rare donors when complicating antibodies arise.

## 1. Introduction

Red blood cell (RBC) alloimmunization, the development of antibodies to foreign antigens from allogeneic transfusions or pregnancy [1], is a potential complication for patients with sickle cell disease (SCD), reflected by an RBC alloimmunization prevalence of 18–37% compared to 2–5% among all transfusion recipients [2]. Higher rates of alloimmunization in SCD are attributed to differences in RBC antigens between donors of European descent and recipients of African descent [3–5]. Due to a high degree of genetic variation in the *RH* locus in persons of African ancestry [6–8], individuals may have Rh variants that lack common epitopes or carry novel epitopes which are not distinguished on RBC antigen serologic phenotyping.

An alloantibody-mediated delayed hemolytic transfusion reaction (DHTR) is an anamnestic response after alloimmunization and subsequent re-exposure to the provoking antigen. Antibodies to donor RBCs may result in life-threatening hemolytic anemia between 24 hours and 28 days after transfusion [9]. Because symptoms can mimic a vaso-occlusive crisis with associated pain and anemia, diagnosis may be delayed in patients with SCD [2]. In the setting of a heightened immune response, patients may also develop new auto- or allo-antibodies [10, 11].

To prevent alloimmunization in patients with SCD, the American Society of Hematology (ASH) guidelines for transfusion in sickle cell suggests phenotypically matching donor units for Rh (C, E or C/c, E/e) and K antigens over only ABO/RhD matching [10]. However, one study found that 45% of chronic and 12% of episodically transfused pediatric patients with SCD were Rh alloimmunized despite serologic D, C, and E antigen matching [6].

We present a pediatric patient with SCD and multiple antibodies, including one to a high prevalence Rh antigen not identified by standard serology, who developed DHTR and describe the significant challenges with antibody identification, the process of obtaining compatible blood, and medical management.

**Figure 1:**
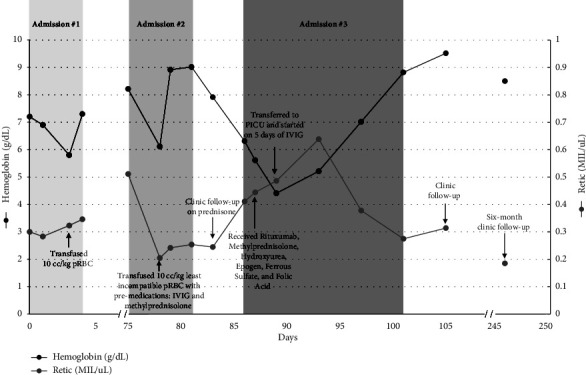
Timeline of patient's clinical course detailing medical interventions with corresponding hemoglobin and reticulocyte count trends.

The case description is detailed below and summarized in Figure 1. Written consent was obtained from the legal guardian of the patient to publish this case report.

## 2. Case Presentation

A 7-year-old girl of African descent with SCD (Hb SS) developed acute chest syndrome (ACS) two days after being admitted for vaso-occlusive pain. She did not have a prior history of transfusions and had a negative antibody screen on admission. This patient's RBC antigen profile had been determined by serologic phenotype at her first hematology clinical encounter one year prior: O Rh(D) Pos C + E- c + e + K- Fy(a-b-) Jk(a-b-) M- S- s+. For management of ACS and severe anemia (hemoglobin [Hb] 5.8 g/dL; baseline 8.7 g/dL), she received 10 mL/kg (230 mL) of O Rh(D) Neg C + E- K- pRBCs, leukoreduced, CMV seronegative, HbS negative. Hemoglobin increased to 7.3 g/dL, and she recovered well and did not require further transfusion. Parents declined to start hydroxyurea based on personal preference. After the hospitalization, she missed multiple scheduled appointments such that follow-up blood counts and antibody screening were not obtained.

Two months later, the patient was admitted to the pediatric intensive care unit for ACS with hypoxemia and respiratory disease. Admission blood screen was positive for an anti-M reactive only at low temperatures and anti-Fy^a^. We recognized the potential for DHTR and thus initiated blood bank work up. Specimen was sent for serologic investigation at the time of presentation. Immunohematology reference laboratory testing later confirmed both anti-M (nonreactive) and anti-Fy^a^, with anti-e “like” antibody. The sample was not sufficient to perform additional testing, and a further specimen was sent for serologic investigation 12 days after presentation. Results showed anti-M, a probable anti-hr^B^ (confirmed by genotyping), and anti-Fya. Additional specialized testing confirmed the serologic antigen typing, except for Jka+ and Jkb+, and with prediction of possible (C)ces haplotype. *RH* genotyping (performed at New York Blood Center) was also sent at the time of this admission and identified the following *RH* allele results: *RHD,* Hybrid *RHD*∗*DIIIa-CE(4–7)-D*, *RHCE*∗*ceS,* and *RHCE*∗*ce(733G).* The predicted RH phenotype was reported as D+, C+(partial), E-, c+(partial), e+(partial), V + VS+, hr^B^-, and hr^S^+.

Due to her worsening clinical status, ACS, and acute drop in hemoglobin to 6.1 g/dL several days into her hospitalization, she was transfused with 10 mL/kg (212 mL) of least incompatible (1+weak) O Rh(D) Pos C- E- K- Fy(a-), leukoreduced, HbS-negative pRBCs with premedication, including methylprednisolone 1 mg/kg q12 h for 2 days, and a single bolus of intravenous immunoglobulin (IVIG) 0.85 g/kg based on ASH 2020 guidelines for prevention of DHTR [10]. Post-transfusion hemoglobin was 8.9 g/dL. Her respiratory status improved, and she was discharged home on prednisone 1 mg/kg twice daily for 5 days, followed by a taper of 0.25 mg/kg every 2 days to prevent rebound vaso-occlusive pain. Parents agreed to start hydroxyurea 20 mg/kg daily to prevent sickle cell-related complications and reduce need for transfusion.

Nine days after her transfusion, she was readmitted for fatigue and jaundice with hemolytic crisis. The hemoglobin was 6.3 g/dL on admission, reticulocyte count was twice baseline, direct antiglobulin test was positive (anti-IgG microscopic, anti-C3 negative), LDH was elevated over 2.5 times the upper limit of normal, and a diagnosis of DHTR was made per ASH definitional criteria [10].

Hemoglobin fractionations (looking for accelerated increase in percentage HbS) were not performed as her hemoglobin dropped below her pretransfusion level (nadir hemoglobin was 4.2 g/dL during that admission), which was consistent with DHTR with hyperhemolysis. Since she did not have other sickle cell complications and remained hemodynamically stable, emergent transfusion was not indicated. Instead, management consisted of hydroxyurea, oral iron and folate supplementation, and erythropoietin 50 units/kg daily to promote erythropoiesis. Given concern for concomitant autoantibody or development of autoantibody [10, 12], immunomodulatory treatment was given with IVIG 0.4 g/kg IV for five days, IV methylprednisolone 1 mg/kg q12 h for 9 days (weaned to IV 0.9 mg/kg q12 h for 3 days and then to oral prednisone 1 mg/kg q12 h on discharge), and rituximab 375 mg/m2 IV weekly for four weeks. She was discharged home to complete steroid wean and continue hydroxyurea (dose escalated to 25 mg/kg). The four-week course of rituximab was completed outpatient. The prednisone was slowly tapered off by 0.25 mg/kg per week over several months to prevent rebound vaso-occlusive pain. The steroid wean was drawn out over a longer course than we intended due to the family's challenge with adherence to follow-up appointments.

Given the results of the serologic and genotype testing, the transfusion recommendation was for crossmatch-compatible O Rh(D) Pos C- E- K- Fy(a-) hr^B^- pRBCs. Two genotype matched antigen negative, nonsickle trait units (C- E- K- Fy(a-) hr^B^-) were obtained from the American Rare Donor Program and retained frozen for potential future transfusion. To date, these have not been required, as she has been well on hydroxyurea during the twelve months of follow-up without vaso-occlusive episodes or acute hemolysis.

## 3. Discussion

Patients with SCD who require transfusions are at a risk of developing DHTR, emphasizing the importance of preventive measures to reduce this risk. We describe a 7-year-old female patient with SCD (Hb SS) with D+, C+(partial), E-, c+(partial), e+(partial), V + VS+, hr^B^-, hr^S^ + RBC phenotype who developed multiple RBC antibodies and suspected DHTR after her second lifetime transfusion, in the setting of severe anemia and ACS. Not only did this patient have an uncommon RH genotype that made it difficult to obtain compatible blood, but also the process of identifying the multiple concurrent RBC antibodies was challenging, requiring a significant volume of blood samples, time, and delay in identification of compatible blood. Although we did not send hemoglobin fractionation as her clinical picture fit with DHTR with hyperhemolysis, looking for a post-transfusion decrease in Hb A and increase in Hb S can confirm the diagnosis.

The antibodies identified in this case were all alloantibodies and included anti-M (cold reacting and only extremely rarely associated with hemolytic transfusion reaction), anti-hr^B^ (generally considered not clinically significant unless transfused with C+ donor cells), and anti-Fy^a^ (associated with mild to severe acute or DHTR). Because this patient received C- and Fy(a-) blood for her second transfusion, anti-hr^B^ and anti-Fy^a^ were considered less likely potential causes of alloantibody-mediated DHTR. Ultimately, the specific antibody that caused our patient's DHTR was not determined. However, given that hyperhemolysis with anti-M has been reported in sickle cell patients [13], it is possible that the anti-M was clinically significant. This case occurred prior to our current practice of routine extended phenotype matching to include Fy(a) for patients with SCD. Our institution performs an extended match for patients who are anticipated to be chronically transfused, which includes C/c, E/e, K, Fya, and S. Since anti-M is usually clinically insignificant, we typically would not recommend matching for patients with anti-M. Instead, we would select the least incompatible units by full crossmatch. However, M-negative units should be considered for patients with sickle cell disease, as this case demonstrates.

Although this patient was successfully managed by transfusion with “least incompatible” O Rh(D) Pos C- E- K- Fy(a-) pRBCs and aggressive medical management including immunosuppressive therapy, erythropoietin, and oral iron per current 2020 ASH clinical guidelines [10], additional measures may assist in the long-term care of SCD patients, who often have complex transfusion requirements. ASH guidelines provide a strong recommendation (based on moderate certainty in the evidence) to prophylactically match red cell antigens for C, E, or C/c, E/e, and K antigens [10]. They suggest that extended red cell antigen matching for Jka/Jkb, Fya/Fyb, M/N, and S/s may provide additional mitigation of alloimmunization and remark that genotyping is preferred over serologic phenotyping given the additional information that it provides, specifically that genotyping can provide increased accuracy for C antigen determination and Fy^b^ antigen matching [4, 10]. As was demonstrated in this case where variant C was detected by specialized testing, extended DNA analysis for RHCE and RHD variants is advisable and can be further informative.

Having RBC genotyping results on file for patients with SCD can facilitate care in two ways—by preventing alloimmunization with potential hemolytic transfusion reaction [14] and by responding rapidly to request rare donors when complicating antibodies arise. With respect to our patient, a prior knowledge of the partial C phenotype could have guided the use of C-donor units to reduce the risk of anti-e “like” or anti-hr^B^ production despite the patient's C+ phenotype. Unaffected family members (i.e., sibling or unaffected 2^nd^ degree family member) can be tested once a proband is identified. If a family member is considered as a donor, then family members without sickle cell trait (HbAS) are preferred. However, in the absence of a family member with HbAA, then an HbAS family member could also be considered with the understanding that there would be less reduction in sickle cell percentage per transfusion. Furthermore, having rare donor programs and including family member participation can facilitate the availability of compatible pRBC products for this patient population. This case has assisted our institution in changing our clinical practice. We now order an RBC genotype on all our patients with SCD at our first encounter with them. Upfront genotyping can contribute to more rapid recovery in critical hemolytic situations for patients with SCD and could be more cost-effective in the long term.

## Data Availability

The case report data on our patient that were used to support the findings of this study are included within the article.
